# Very Low and High Levels of Vitamin D Are Associated with Shorter Leukocyte Telomere Length in 148,321 UK Biobank Participants

**DOI:** 10.3390/nu15061474

**Published:** 2023-03-19

**Authors:** Chia-Ling Kuo, Ben Kirk, Meiruo Xiang, Luke C. Pilling, George A. Kuchel, Richard Kremer, Gustavo Duque

**Affiliations:** 1 Connecticut Convergence Institute for Translation in Regenerative Engineering, University of Connecticut Health, Farmington, CT 06030, USA; 2 Center on Aging, University of Connecticut Health, Farmington, CT 06030, USA; 3 Department of Medicine, Western Health, Melbourne Medical School, University of Melbourne, St Albans, Melbourne, VIC 3021, Australia; 4 Australian Institute for Musculoskeletal Science (AIMSS), University of Melbourne and Western Health, St Albans, Melbourne, VIC 3021, Australia; 5 Epidemiology and Public Health Group, Faculty of Health and Life Sciences, University of Exeter, Exeter EX4 4PY, UK; 6 Research Institute of the McGill University Health Centre, Montreal, QC H4A 3J1, Canada; 7 Dr. Joseph Kaufmann Chair in Geriatric Medicine, Department of Medicine, McGill University, Montreal, QC H4A 3J1, Canada

**Keywords:** telomerase, leukocyte telomere length, vitamin D, aging, serum levels, geroscience

## Abstract

**Background:** Shorter leukocyte telomere length (LTL) is observed in multiple age-related diseases, which are also associated with vitamin D deficiency (i.e., osteosarcopenia, neurocognitive disorders, cancer, osteoarthritis, etc.), suggesting a close association between vitamin D and LTL. In this study, we examined the relationship between vitamin D levels and LTL in older participants of the UK Biobank. **Methods:** Data were collected from the UK Biobank. Participants aged 60 and older (*n* = 148,321) were included. Baseline LTL was measured using a multiplex qPCR technique and expressed as the ratio of the telomere amplification product (T) to that of a single-copy gene (S) (T/S ratio). Serum 25-hydroxyvitamin D (25OHD) was stratified by z score and linked to LTL in a linear regression model adjusting for covariates. **Results:** Compared to the medium level, a low (in the range of 16.6 nmol/L, 29.7 nmol/L) or extremely low (≤16.6 nmol/L) level of serum 25OHD was associated with shorter LTL: 0.018 SD (standardized β = −0.018, 95% CI −0.033 to −0.003, *p* = 0.022) and 0.048 SD (standardized β = −0.048, 95% CI −0.083 to −0.014, *p* = 0.006), respectively. Additionally, the high serum 25OHD groups (>95.9 nmol/L) had 0.038 SD (standardized β = −0.038, 95% CI −0.072 to −0.004, *p* = 0.030) shorter mean LTL than the group with medium 25OHD levels. The associations above were adjusted for multiple variables. **Conclusions:** In this population-based study, we identified an inverted U-shape relationship between LTL and vitamin D status. Our findings could be affected by unmeasured confounders. Whether high or low vitamin D-associated shorter LTL is mechanistically related to age-related conditions remains to be elucidated.

## 1. Introduction

Genome instability is considered one of the hallmarks of aging [1]. Telomeres are one of several key elements required for genome stability. Telomeric DNA consists of tandem repeats of a simple, often G-rich, sequence. This sequence is determined by the action of telomerase, which lengthens terminal regions of eukaryotic telomeric DNA by RNA-templated addition of the repeated DNA sequence [2]. However, with advancing age, telomerase activity is affected, and telomeres start shortening, which compromises cell function and lifespan [2]. This shortening has been associated with multiple age-related diseases, including cardiovascular disease, malignancies, dementia, osteosarcopenia, frailty, and other conditions [2,3,4].

Vitamin D is a micronutrient with an important role in inflammation, cell growth, differentiation, and apoptosis [5]. Vitamin D insufficiency, defined as serum levels of 25-hydroxyvitamin D [25(OH)D] concentrations below 50 nmol/L, has also been associated with the age-related diseases listed above [6]. Therefore, a link between serum 25OHD levels and telomere shortening has been proposed but remains partially explored [7,8]. In a recent study analyzing data from 1542 younger adults (aged 20–39 years), 1336 middle-aged adults (aged 40–59 years), and 1382 older adults (aged ≥ 60 years) participants in the US NHANES 2001–2002, Beilfuss et al. [9] reported that serum 25(OH)D was positively associated with leukocyte telomere length (LTL) in middle-aged participants (aged 40–59 years) only, independently of other factors. However, this association was discrete and not observed in older participants. Liu et al. [10] examined the cross-sectional association between serum 25OHD concentration in plasma and LTL in 1154 US radiologic technologists aged 48–93 (373 white females, 278 white males, 338 black females, 165 black males) and found a weak positive association between 25(OH)D and LTL over the entire range of 25(OH)D levels in the overall study population and subgroups as a function of sex and race. Other studies have shown conflicting results and are difficult to interpret because of their small sample size [2].

In addition to the deleterious effect of vitamin D deficiency and its known association with multiple age-related conditions [5], the impact of very high 25OHD serum levels on the pathogenesis of those conditions remains to be elucidated. Recent research has identified a “U-shaped association” with morbidity and mortality risks at both high and low 25 (OH) D levels [11,12]. However, the underlying mechanisms explaining the harmful effects of having too much or too little circulating vitamin D levels remain elusive. Interestingly, there is growing evidence suggesting that higher circulating vitamin D levels are associated with longer LTL, thus having a potentially beneficial effect on aging and age-related diseases [2]. If this hypothesis proved to be correct, longer LTL, induced in part by vitamin D, would be responsible for improved cell differentiation, function and survival, and overall healthy aging. To test this hypothesis, the present study examined the relationship between 25OHD levels and LTL in the very large and well-established UK Biobank.

## 2. Materials and Methods

### 2.1. UK Biobank

Over 500,000 participants aged 40–70 years were recruited between 2006 and 2010. Participants visited one of 22 assessment centers near their residences. Baseline assessments (at recruitment) included physical measures, such as grip strength, heel ultrasound, and bioimpedance measurements, biological samples (blood, saliva, and urine samples) for various assays, and surveys on demographics, lifestyle, and environmental factors, plus personal and family medical history [13]. UK Biobank received ethical approval from the Northwest Centre for Research Ethics Committee (11/NW/0382), and all participants provided written informed consent.

### 2.2. Data

Data used to examine the association between LTL and serum 25OHD included: (1) LTL from the baseline visit; (2) 25OHD levels at the baseline visit; (3) baseline covariates: serum calcium; anthropometric; demographic or socioeconomic variables; and lifestyle factors. The UK Biobank field IDs used to find the data above are provided in Table 1.

### 2.3. Inclusion and Exclusion Criteria

**Figure 1:**
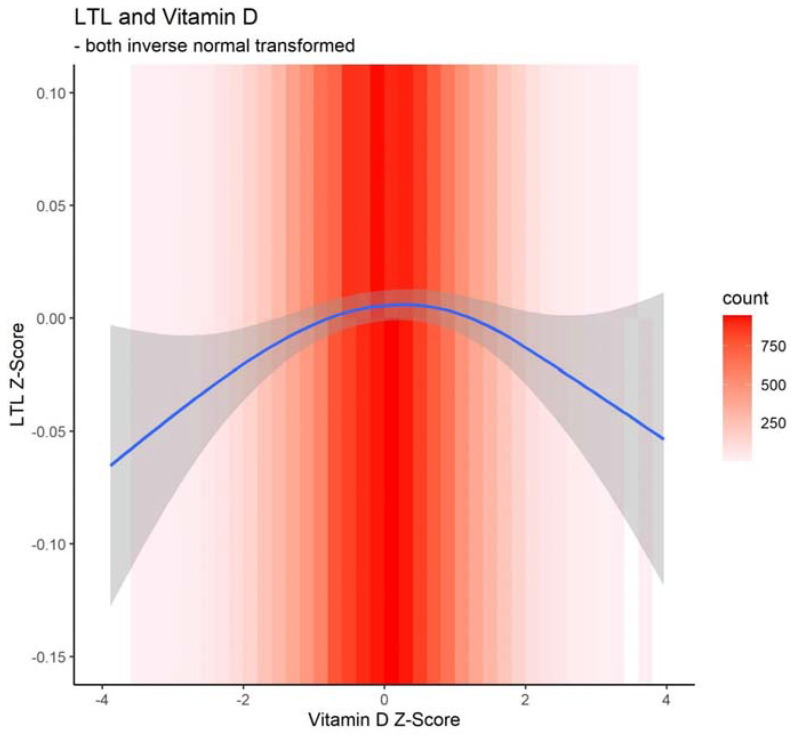
Flowchart of the final analytical sample.

UK Biobank participants attending the baseline visit were included regardless of self-reported ethnicities; we chose 60 years as a cut-off due to the high prevalence and clinical significance of vitamin D deficiency in this population [5,7]. Additionally, participants with any missing data were excluded, leaving a total of 148,321 participants for analysis (Figure 1).

### 2.4. Leukocyte Telomere Length

DNA was extracted from peripheral blood leukocytes. LTL was measured using a multiplex qPCR-based technique by comparing the amount of the telomere amplification product (T) to that of a single-copy gene (S). A T/S ratio was derived, representing the mean LTL. LTL adjusted for the influence of technical parameters was released by UK Biobank and used in this project.

### 2.5. Defining Serum Vitamin D Status

Biochemical assays were performed on blood samples collected during the baseline evaluation at the assessment centers. Samples were collected in a silica clot accelerator tube and stored at −80 °C. These samples were later processed in a central laboratory using an automated dispensing system [14]. Serum 25(OH)D status was measured by chemiluminescence immunoassay (DiaSorin LIAISON XL, Gerenzano, Italy), which was certified by the Vitamin D Standardization Certification Program of the Centers for Disease Control and Prevention [15]. To ensure the precision of analysis, quality control samples at different concentrations were analyzed [16], and the accuracy of 25OHD was verified through the RIQAS Immunoassay Specialty I EQA program (Randox Laboratories, Kearneysville, West Virginia, USA), an external quality assurance scheme [17].

### 2.6. Covariates

Race included White, Black, South Asian, and Other ethnicities. Education ranged from none to college or university degree (higher education). Townsend deprivation index was a measure of material deprivation at the postcode level based on the preceding national census data (mean 0 in the UK population), with higher scores representing greater levels of deprivation. Whole body fat mass was measured via the bioelectrical impedance analysis. Smoking status (never, previous, or current) was accessed via a touchscreen questionnaire, and similarly, for alcohol intake frequency (daily or almost daily, three or four times a week, once or twice a week, one to three times a month, special occasions only). Physical activity was assessed by adapted questions from the short International Physical Activity Questionnaire (IPAQ) [18]. Time spent in vigorous, moderate, and walking activities was weighted by their intensity levels to derive the total metabolic equivalent task (MET) minutes per week, which, along with days of each activity for a certain duration, were used to determine low, moderate, or high physical activity level, following the IPAQ guidelines. Serum calcium was measured using a Beckman Coulter (UK) Ltd. assay and Beckman Coulter AUS800 platform via colorimetric analysis methodology. Units of measurement were mmol/L, and the manufacturer’s analytical range was 1–3.5 mmol/L. Extensive QC procedures were followed to identify invalid results, dilution issues, and laboratory drift, as previously described [17]. For sensitivity analysis, we included the season of assessment, which has been known to be associated with vitamin D levels, to evaluate the robustness of the association between 25OHD and LTL.

### 2.7. Statistical Methods

Serum 25OHD was linked to LTL in a linear regression model adjusting for covariates. Serum 25OHD and LTL were z-transformed using the rank-based inverse normal transformation prior to the association analysis. The transformed 25OHD and LTL followed a standard normal distribution with a mean of 0 and a standard deviation of 1. To capture a non-linear relationship of 25OHD with LTL, 25OHD was categorized into five groups with z-scores in the ranges of ≤−2, (−2, −1], (−1, 1] (reference), (1, 2], and >2, corresponding to the ranges of 25OHD in the original scale: ≤16.6 nmol/L; (16.6 nmol/L, 29.7 nmol/L]; (29.7 nmol/L, 71.8 nmol/L] (reference); (71.8 nmol/L, 95.9 nmol/L]; and >95.9 nmol/L. For convenience, the five 25OHD groups were named as follows: extremely low; low; medium; moderately high; and high. The unadjusted associations between 25OHD and LTL were reported, as well as associations adjusting for demographic/socioeconomic variables (age, sex, ethnicity, Townsend deprivation index, education), whole body fat mass (z-transformed by the rank-based inverse normal distribution), and lifestyle factors (smoking status, alcohol intake frequency, and IPAQ activity group), and serum calcium (z-transformed by the rank-based inverse normal transformation). All *p*-values smaller than 5% were considered statistically significant. All the statistical analyses were performed in R version 4.1.2 (https://www.r-project.org/; accessed on 17 March 2023).

## 3. Results

### 3.1. Population Characteristics

Data were obtained at the baseline visit when the mean age of the included samples was 64.13 years (SD: 2.85). Fifty percent of participants were women, and the vast majority were of European ancestry (97.3%). A total of 27.5% of the participants received a college or university degree, whereas 25.5% had no degree. Overall, according to the Townsend deprivation index, the included samples were less materially deprived than the population on average. Fifty percent had never smoked, 92% drank more or less, and 86.3% reported moderate to high physical activity levels (Table 2).

### 3.2. Associations between Telomere Length and Serum 25OHD

**Figure 2:**
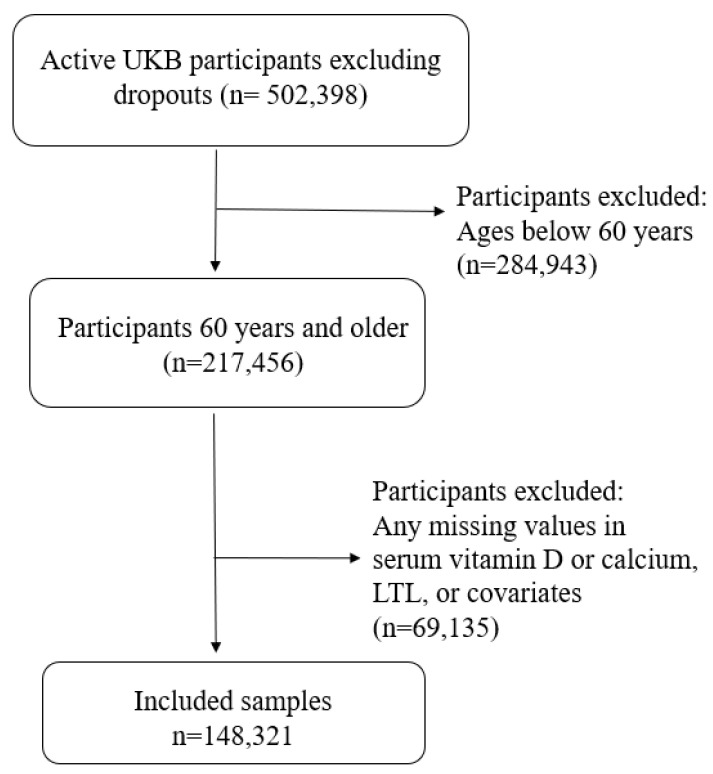
Relationship of z-transformed leukocyte telomere length versus z-transformed vitamin D (curve plotted based on the generalized additive model including a cubic regression spline with shrinkage-to-zero).

As shown in Figure 2, there was an inverted U-shaped relationship between z-transformed 25OHD and LTL. Low and extremely low 25OHD levels were significantly associated with shorter LTL as well as high 25OHD levels in both unadjusted and adjusted association analyses (Table 3). In the adjusted association analysis (Table 3), a low (z score in (−1, 1] or serum 25OHD in (29.7 nmol/L, 71.8 nmol/L]) or extremely low (z score ≤ −2 or serum 25OHD ≤ 16.6 nmol/L) level of serum 25OHD compared to the medium level (z score in (−1, 1] or serum 25OHD in (29.7 nmol/L, 71.8 nmol/L]) was associated with shorter LTL: 0.018 SD (standardized β = −0.018, 95% CI −0.033 to −0.003, *p* = 0.022) and 0.048 SD (standardized β = −0.048, 95% CI −0.083 to −0.014, *p* = 0.006) shorter mean LTL, respectively. Additionally, the high serum 25OHD group (z score > 2 or serum 25OHD > 95.9 nmol/L) had 0.038 SD shorter mean LTL than the group with medium 25OHD levels (z score in (−1, 1] or serum 25OHD in (29.7 nmol/L, 71.8 nmol/L]). As expected, the adjusted model (Table 3) showed that older age and males were associated with shorter LTL. Longer LTL was observed in Blacks than in Whites and South Asians. Higher education, lower whole body fat mass, never smoking, and physical activity was associated with longer LTL, but lower Townsend deprivation or alcohol intake frequency was not significantly associated with LTL. Calcium in the blood also was not significantly associated with LTL. While the mean 25OHD level was lower in spring (45.92 ± 19.78 nmol/L) and winter (44 ± 19.33 nmol/L) than in summer (59.19 ± 19.48 nmol/L) and fall (55.37 ± 19.98 nmol/L), including 25OHD in the model for LTL, minimally changed the association between 25OHD and LTL (standardized β associated with extremely low 25OHD [versus medium 25OHD] = −0.046, 95% CI −0.081 to −0.011, *p* = 0.010; standardized β associated with low 25OHD [versus medium 25OHD] = −0.016, 95% CI −0.031 to −0.001, *p* = 0.043; standardized β associated with moderate 25OHD [versus medium 25OHD] = −0.003, 95% CI −0.018 to 0.012, *p* = 0.693; standardized β associated with high 25OHD [versus medium 25OHD] = −0.039, 95% CI −0.073 to −0.005, *p* = 0.026).

## 4. Discussion

In this population-based study, we examined the association between LTL and vitamin D status. Shorter LTL was associated with high and low 25OHD levels in older individuals aged 60 and older. Interestingly, we found an inverted U-shaped association between shorter LTL and serum 25OHD at very low and high serum levels.

The clinical implications of vitamin D deficiency on the musculoskeletal system [6], which include osteosarcopenia [19] and rheumatic conditions [20], are well-established. In addition, vitamin D deficiency is also associated with the development of age-related conditions such as Alzheimer’s disease, Parkinson’s disease, multiple sclerosis, and cardiovascular disease [6]. Although the biological mechanisms explaining these associations remain partially elucidated, there is mounting evidence to propose that normal vitamin D levels are required to delay the appearance of several of the major biological hallmarks of aging [1,6], including genomic instability, telomere attrition, epigenetic alterations, loss of proteostasis, disabled autophagy, mitochondrial dysfunction, cellular senescence, stem cell exhaustion, and chronic inflammation [6,21].

Amongst these mechanisms, the effect of vitamin D on telomere attrition has received significant attention not only because of its biological plausibility but also because there is a clear correlation between those age-related conditions associated with vitamin D deficiency and those associated with telomere shortening [2,6]. Vitamin D may reduce telomere shortening through anti-inflammatory and anti-cell proliferation mechanisms. Although most of these mechanisms have been tested in cancer cells, vitamin D has been found to play an important role in regulating the telomerase reverse transcriptase (hTERT) promoter via small non-coding RNA molecules [22]. Regarding targeting this association in humans, only a few clinical trials have tested the effect of vitamin D supplementation on telomere length in vitamin D-deficient populations, and the reports appear contradictory. Yang et al. [23] tested the effect of vitamin D supplementation on cognitive function in 183 subjects randomized to an intervention group (vitamin D 800 IU/day, *n* = 93) or a placebo group (the matching starch granules, *n* = 90), and followed up for 12 months. They reported that vitamin D supplementation for 12 months improved cognitive function by reducing oxidative stress regulated by increased LTL in older adults with mild cognitive impairment. In contrast, Agirbasli et al. [24] investigated the short-term effects of vitamin D supplementation on LTL in a cohort of vitamin D-deficient postmenopausal women (*n* = 102). The group was divided into supplementation those with oral vitamin D_3_ (cholecalciferol) at a dose of 50,000 IU/week for eight weeks (*n* = 52) and placebo groups (*n* = 50). At the end of the study period, LTL levels were significantly increased in both groups, and this change was more prominent in the placebo group.

Although normalization of serum 25OHD levels in older individuals could have a beneficial effect on LTL and, thus, partially explain the therapeutic impact of vitamin D supplementation on age-related conditions, there is always a risk of administering too much supplementation and inducing harmful effects associated with high serum levels of vitamin D. The finding of an inverted U-shaped curve in this study is very relevant since it has been previously associated with adverse outcomes. Indeed, hypo- or hypervitaminosis D in mice causes accelerated aging and has shown a U-shaped association between serum 25(OH)2D and the risk of cancer in those models [22,25]. In humans, results from observational, population-based studies and randomized clinical trials have shown a U- or J-shaped curve and suggested an increased risk of adverse outcomes in those with the highest serum 25OHD levels, including falls, fractures, and frailty [26,27,28]. Most studies have reported a higher risk in those participants with serum levels of 25OHD above 100 nmol/L. Although these findings have discouraged the practice of administering high-loading doses of vitamin D, the mechanisms underlying the negative impact of high serum levels of 25OHD remain mostly speculative [28]. Since telomere shortening is also associated with an increased risk of these adverse events [29,30], it is, therefore, tempting to speculate that induction of LTL shortening by high levels of serum 25OHD could be among the involved mechanisms but require further studies. Alternatively, a biologically plausible mechanism is that a high level of 25OHD (which could be a result of different regimens) may have triggered a short-term “protective” reaction in which CYP24 (25-hydroxyvitamin D-24-hydroxylase), the enzyme that catabolizes 1,25-dihydroxy vitamin D was up-regulated, resulting in decreased blood and tissue levels of 1,25-dihydroxy vitamin D [31].

In the present study, our data show a non-linear relationship between 25OHD and LTL after modeling vitamin D by the serum 25OHD z-score groups: ≤−2, (−2, −1], (−1, 1], (1, 2], and >2. A z score of −2 corresponds to serum 25OHD 16.6 nmol/L in the original scale, and a z score of 2 corresponds to 95.9 nmol/L. These extremely low and moderately high 25OHD serum levels approach those previously associated with adverse outcomes in clinical trials (26). Whether a dose response could explain these findings goes beyond the scope of this study.

## 5. Conclusions

In conclusion, our study is the first of its kind to demonstrate an inverted U-shape relationship between LTL and vitamin D status in a large sample of community-dwelling individuals. We conducted this analysis by adjusting for various demographic/socioeconomic variables and lifestyle factors known to affect serum vitamin D and/or telomere length. We also controlled for whole body fat since obese and overweight individuals tend to demonstrate blunted responses to vitamin D replacement [32]. A limitation of this study is the healthy volunteer selection bias, which is alleviated by significant heterogeneity of exposure measures [33]. Additionally, the cross-sectional nature of our study prohibits any causal inferences, limiting our ability to exclude the role of unknown confounders or the existence of reverse causation. Nevertheless, non-linear Mendelian randomization methods are useful tools to test causality [34]. Further mechanistic studies in animal models and human subjects are still required.

## Figures and Tables

**Table 1:** Data used to examine the association between leukocyte telomere length(LTL) and serum 25OHD. https://biobank.ndph.ox.ac.uk/showcase/search.cgi (accessed on 17 March 2023).

Variable	UK Biobank Data Field
Leukocyte telomere length (T/S ratio), adjusted for the influence of technical parameters	22,191
25OHD (nmol/L)	30,890
Calcium (mmol/L)	30,680
Age	21,003
Sex	31
Ethnicity	21,000
Education	6138
Townsend deprivation index at recruitment	189
Whole body fat mass	23,100
Smoking status	20,116
Alcohol intake frequency	1558
International Physical Activity Questionnaire (IPAQ) activity group	22,032

**Table 2:** Participant characteristics of the included samples.

Variable	Mean ± SD (Min, Max) or Frequency (%)
Leukocyte telomere length (T/S ratio), adjusted for the influence of technical parameters	0.81 ± 0.12; (0.08, 3.72)
Calcium (mmol/L)	2.38 ± 0.09; (1.19, 3.57)
Vitamin D (nmol/L)	51.34 ± 20.65; (10, 276)
Extremely low ^1^	3333 (2.2%)
Low ^2^	20,191 (13.6%)
Medium ^3^ (reference)	101,195 (68.2%)
Moderately high ^4^	20,215 (13.6%)
High ^5^	3387 (2.3%)
Age (years)	64.13 ± 2.85; (60, 70)
Sex	
Female	74,549 (50.3%)
Male	73,772 (49.7%)
Ethnicity	
White	144,292 (97.3%)
Black	1048 (0.7%)
South Asian	1655 (1.1%)
Other	1326 (0.9%)
Townsend deprivation index	−1.62 ± 2.92; (−6.26, 10.59)
Education	
None	37,779 (25.5%)
CSEs	2677 (1.8%)
GCSEs/O-levels	20,977 (14.1%)
A-levels/NVQ/HND/HNC	22,521 (15.2%)
Prof. qualification (e.g., nursing, teaching)	23,632 (15.9%)
College or University degree	40,735 (27.5%)
Whole body fat mass (kg)	24.9 ± 8.84; (5, 108.1)
Smoking status	
Never	73,929 (49.8%)
Current	11,739 (7.9%)
Previous	62,653 (42.2%)
Alcohol intake frequency	
Never	11,865 (8%)
Special occasions only	16,934 (11.4%)
One to three times a month	14,470 (9.8%)
Once or twice a week	35,499 (23.9%)
Three or four times a week	33,689 (22.7%)
Daily or almost daily	35,864 (24.2%)
IPAQ group	
Low	20,213 (13.6%)
Moderate	67,683 (45.6%)
High	60,425 (40.7%)
Season of assessment	
Spring	43,634 (29.4%)
Summer	39,583 (26.7%)
Fall	35,501 (23.9%)
Winter	29,603 (20.0%)

^1^ **extremely low**: z score ≤ −2 or serum 25OHD ≤ 16.6 nmol/L; ^2^ **low**: z score in (−2, −1] or serum 25OHD in (16.6 nmol/L, 29.7 nmol/L]; ^3^ **medium**: z score in (−1, 1] or serum 25OHD in (29.7 nmol/L, 71.8 nmol/L]; ^4^ **moderately high**: z score in (1, 2] or serum 25OHD in (71.8 nmol/L, 95.9 nmol/L]; **^5^ high**: z score >2 or 25OHD > 95.9 nmol/L.

**Table 3:** Association between vitamin D and leukocyte telomere length, unadjusted and adjusted for covariates.

		95% CI			
Model	Beta	Lower	Upper	*p*-Value	Overall *p*-Value
Unadjusted					
Vitamin D					<0.001
Extremely low ^1^	−0.053	−0.088	−0.019	0.002	
Low ^2^	−0.018	−0.033	−0.003	0.021	
Medium ^3^ (reference)					
Moderately high ^4^	−0.003	−0.018	0.012	0.663	
High ^5^	−0.051	−0.086	−0.017	0.003	
Adjusted					
Vitamin D					0.004
Extremely low ^1^	−0.048	−0.083	−0.014	0.006	
Low ^2^	−0.018	−0.033	−0.003	0.022	
Medium ^3^ (reference)					
Moderately high ^4^	−0.002	−0.017	0.013	0.778	
High ^5^	−0.038	−0.072	−0.004	0.030	
Serum calcium (z score)	−0.0004	−0.006	0.005	0.869	0.869
Age (years)	−0.030	−0.032	−0.028	1.44 × 10^−234^	<0.001
Sex					<0.001
Female	Ref	Ref	Ref	Ref	
Male	−0.220	−0.231	−0.209	<2.2 × 10^−16^	
Ethnicity					<0.001
White	Ref	Ref	Ref	Ref	
Black	0.363	0.302	0.424	1.23 × 10^−31^	
South Asian	−0.010	−0.059	0.039	0.695	
Other	0.182	0.129	0.236	3.01 × 10^−11^	
Townsend deprivation index	−0.001	−0.003	0.001	0.344	0.344
Education					<0.001
None	Ref	Ref	Ref	Ref	
CSEs	0.012	−0.027	0.051	0.555	
GCSEs/O-levels	0.035	0.018	0.052	5.02 × 10^−5^	
A-levels/NVQ/HND/HNC	0.032	0.015	0.048	1.80 × 10^−4^	
Prof. qualification (e.g., nursing, teaching)	0.058	0.042	0.075	3.35 × 10^−12^	
College or University degree	0.097	0.082	0.111	6.49 × 10^−39^	
Whole body fat mass	−0.026	−0.032	−0.021	1.65 × 10^−21^	<0.001
Smoking status					<0.001
Never	Ref	Ref	Ref	Ref	
Current	−0.095	−0.115	−0.075	3.32 × 10^−21^	
Previous	−0.024	−0.035	−0.013	1.72 × 10^−5^	
Alcohol intake frequency					0.714
Never	Ref	Ref	Ref	Ref	
Special occasions only	0.002	−0.021	0.025	0.856	
One to three times a month	0.006	−0.018	0.031	0.602	
Once or twice a week	0.000	−0.021	0.021	0.982	
Three or four times a week	−0.007	−0.028	0.014	0.518	
Daily or almost daily	−0.007	−0.028	0.015	0.550	
IPAQ group					0.010
Low	Ref	Ref	Ref	Ref	
Moderate	0.024	0.008	0.039	0.003	
High	0.021	0.005	0.037	0.009	

^1^ **extremely low**: z score ≤ −2 or serum 25OHD ≤ 16.6 nmol/L; ^2^ **low**: z score in (−2, −1] or serum 25OHD in (16.6 nmol/L, 29.7 nmol/L]; ^3^ **medium**: z score in (−1, 1] or serum 25OHD in (29.7 nmol/L, 71.8 nmol/L]; ^4^ **moderately high**: z score in (1, 2] or serum 25OHD in (71.8 nmol/L, 95.9 nmol/L]; **^5^ high**: z score > 2 or serum 25OHD > 95.9 nmol/L.

## Data Availability

The datasets generated during and/or analyzed during the current study are available from the corresponding author upon reasonable request.

## References

[1] López-Otín C., Blasco M.A., Partridge L., Serrano M., Kroemer G. (2023). Hallmarks of aging: An expanding universe. Cell.

[2] Gruber H.J., Semeraro M.D., Renner W., Herrmann M. (2021). Telomeres and Age-Related Diseases. Biomedicines.

[3] Kirk B., Al Saedi A., Duque G. (2019). Osteosarcopenia: A case of geroscience. Aging Med..

[4] Lorenzi M., Bonassi S., Lorenzi T., Giovannini S., Bernabei R., Onder G. (2018). A review of telomere length in sarcopenia and frailty. Biogerontology.

[5] Umar M., Sastry K.S., Chouchane A.I. (2018). Role of Vitamin D Beyond the Skeletal Function: A Review of the Molecular and Clinical Studies. Int. J. Mol. Sci..

[6] Berridge M.J. (2017). Vitamin D deficiency accelerates ageing and age-related diseases: A novel hypothesis. J. Physiol..

[7] Zarei M., Zarezadeh M., Hamedi Kalajahi F., Javanbakht M.H. (2021). The Relationship Between Vitamin D and Telomere/Telomerase: A Comprehensive Review. J. Frailty Aging.

[8] Mazidi M., Mikhailidis D.P., Banach M., Dehghan A. (2020). Impact of serum 25-hydroxyvitamin D 25(OH) on telomere attrition: A Mendelian Randomization study. Clin. Nutr..

[9] Beilfuss J., Camargo C.A., Kamycheva E. (2017). Serum 25-Hydroxyvitamin D Has a Modest Positive Association with Leukocyte Telomere Length in Middle-Aged US Adults. J. Nutr..

[10] Liu J.J., Cahoon E.K., Linet M.S., Little M.P., Dagnall C.L., Higson H., Savage S.A., Freedman D.M. (2016). Relationship between plasma 25-hydroxyvitamin D and leucocyte telomere length by sex and race in a US study. Br. J. Nutr..

[11] Grant W.B., Karras S.N., Bischoff-Ferrari H.A., Annweiler C., Boucher B.J., Juzeniene A., Garland C.F., Holick M.F. (2016). Do studies reporting ‘U’-shaped serum 25-hydroxyvitamin D-health outcome relationships reflect adverse effects?. Dermato-Endocrinology.

[12] Davis C.D. (2009). Vitamin D and health: Can too much be harmful?. Am. J. Lifestyle Med..

[13] Sudlow C., Gallacher J., Allen N., Beral V., Burton P., Danesh J., Downey P., Elliott P., Green J., Landray M. (2015). UK biobank: An open access resource for identifying the causes of a wide range of complex diseases of middle and old age. PLoS Med..

[14] Elliott P., Peakman T.C., UK Biobank (2008). The UK Biobank sample handling and storage protocol for the collection, processing and archiving of human blood and urine. Int. J. Epidemiol..

[15] CDC CDC Vitamin D Standardization-Certification Program (CDC VDSCP).

[16] UK Biobank (2019). Biomarker Assay Quality Procedures: Approaches Used to Minimise Systematic and Random Errors.

[17] UK Biobank (2019). Companion Document for Serum Biomarker Data.

[18] Cassidy S., Chau J.Y., Catt M., Bauman A., Trenell M.I. (2016). Cross-sectional study of diet, physical activity, television viewing and sleep duration in 233,110 adults from the UK Biobank; the behavioural phenotype of cardiovascular disease and type 2 diabetes. BMJ Open.

[19] Bruyère O., Cavalier E., Reginster J.Y. (2017). Vitamin D and osteosarcopenia: An update from epidemiological studies. Curr. Opin. Clin. Nutr. Metab. Care.

[20] Charoenngam N. (2021). Vitamin D and Rheumatic Diseases: A Review of Clinical Evidence. Int. J. Mol. Sci..

[21] Giudici K.V. (2021). Nutrition and the Hallmarks of Aging. J. Nutr. Health Aging.

[22] Pusceddu I., Farrell C.J., Di Pierro A.M., Jani E., Herrmann W., Herrmann M. (2015). The role of telomeres and vitamin D in cellular aging and age-related diseases. Clin. Chem. Lab. Med..

[23] Yang T., Wang H., Xiong Y., Chen C., Duan K., Jia J., Ma F. (2020). Vitamin D Supplementation Improves Cognitive Function Through Reducing Oxidative Stress Regulated by Telomere Length in Older Adults with Mild Cognitive Impairment: A 12-Month Randomized Controlled Trial. J. Alzheimer’s Dis..

[24] Agirbasli D., Kalyoncu M., Muftuoglu M., Aksungar F.B., Agirbasli M. (2022). Leukocyte telomere length as a compensatory mechanism in vitamin D metabolism. PLoS ONE.

[25] Tuohimaa P. (2009). Vitamin D and aging. J. Steroid Biochem. Mol. Biol..

[26] Sanders K.M., Stuart A.L., Williamson E.J., Simpson J.A., Kotowicz M.A., Young D., Nicholson G.C. (2010). Annual high-dose oral vitamin D and falls and fractures in older women: A randomized controlled trial. JAMA.

[27] Kojima G., Iliffe S., Tanabe M. (2017). Vitamin D supplementation as a potential cause of U-shaped associations between vitamin D levels and negative health outcomes: A decision tree analysis for risk of frailty. BMC Geriatr..

[28] Sanders K.M., Nicholson G.C., Ebeling P.R. (2013). Is high dose vitamin D harmful?. Calcif. Tissue Int..

[29] Wong S.K., Ima-Nirwana S., Chin K.Y. (2020). Can telomere length predict bone health? A review of current evidence. Bosn. J. Basic Med. Sci..

[30] Rippberger P.L., Emeny R.T., Mackenzie T.A., Bartels S.J., Batsis J.A. (2018). The association of sarcopenia, telomere length, and mortality: Data from the NHANES 1999–2002. Eur. J. Clin. Nutr..

[31] Dawson-Hughes B., Harris S.S. (2010). High-dose vitamin D supplementation: Too much of a good thing?. JAMA.

[32] Tobias D.K., Luttmann-Gibson H., Mora S., Danik J., Bubes V., Copeland T., LeBoff M.S., Cook N.R., Lee I.M., Buring J.E. (2023). Association of Body Weight With Response to Vitamin D Supplementation and Metabolism. JAMA Netw. Open.

[33] Fry A., Littlejohns T.J., Sudlow C., Doherty N., Adamska L., Sprosen T., Collins R., Allen N.E. (2017). Comparison of Sociodemographic and Health-Related Characteristics of UK Biobank Participants With Those of the General Population. Am. J. Epidemiol..

[34] Staley J.R., Burgess S. (2017). Semiparametric methods for estimation of a nonlinear exposure-outcome relationship using instrumental variables with application to Mendelian randomization. Genet. Epidemiol..

